# Creation and Characterization of a Breast Cancer Tissue Microarray Including Black and White Patients from Florida and Hispanic Patients from Puerto Rico and Florida

**DOI:** 10.1158/2767-9764.CRC-24-0650

**Published:** 2025-05-16

**Authors:** Abigail E. Lantz, Edna R. Gordián, Marilin Rosa, Marileana Rodríguez-Ruíz, Joseph O. Johnson, Ryan Gebert, Allison Bahr, Dung Tsa Chen, Julie Dutil, Jiannong Li, José A. Oliveras Torres, Harold I. Saavedra, Steven A. Eschrich, Idhaliz Flores, William D. Cress

**Affiliations:** 1 Department of Molecular Oncology, H. Lee Moffitt Cancer Center & Research Institute, Tampa, Florida.; 2 Department of Pathology, H. Lee Moffitt Cancer Center & Research Institute, Tampa, Florida.; 3 Medical Doctor Program, Ponce Health Sciences University, Ponce, Puerto Rico.; 4 Analytic Microscopy Core, H. Lee Moffitt Cancer Center & Research Institute, Tampa, Florida.; 5 Department of Biostatistics and Bioinformatics, H. Lee Moffitt Cancer Center & Research Institute, Tampa, Florida.; 6 Department of Basic Sciences, Ponce Research Institute, Ponce Health Sciences University, Ponce, Puerto Rico.

## Abstract

Breast cancer is a leading cause of cancer-related mortality among women worldwide and is known to have higher mortality among women with African ancestry. Herein, we describe the creation and characterization of a multiethnic breast cancer tissue microarray (ME-BrTMA) representing tumors from non-Hispanic White (*n* = 41), non-Hispanic Black (NHB; *n* = 45), and Hispanic patients from Puerto Rico (*n* = 36) and Florida (*n* = 52). This ME-BrTMA comprises five blocks with a total of 610 cores: 371 breast cancer tumor cores, 93 breast stromal cores, 96 normal breast tissue cores, 30 non–breast cancer tumor cores, and 20 cores representing normal tissues. Initial characterization of the ME-BrTMA includes standard IHC staining of well-characterized clinical biomarkers, including the estrogen hormone receptors and progesterone hormone receptors, HER2, and Ki-67, interpreted by the coauthoring pathologist (Marilin Rosa). The IHC results indicated good but imperfect alignment with clinical diagnoses. Cores from breast cancer tumors from the NHB cohort most frequently scored negative for estrogen receptor (63%, *P* < 0.005) and progesterone receptor (80%, *P* < 0.005) and most frequently have high expression of the Ki-67 proliferation marker (38%, *P* < 0.05). Prediction Analysis of Microarray 50 (PAM50) analysis using RNA from secondary patient blocks showed that the NHB group also most frequently scored in the basal-like category (61%, *P* < 0.05). Taken together, the initial characterization of the ME-BrTMA suggests that it may serve as a representative resource to understand the underlying biology of breast cancer and its relationship to patient outcomes.

**Significance:**

The ME-BrTMA described herein provides a resource that may serve as a tool to understand the underlying biology of breast cancer.

## Introduction

The racial and ethnic diversity in cancer research studies including large sequencing efforts (1–6), clinical trials (7–10), and banked tissue resources (11–15) is limited despite evidence that many underrepresented groups, including Hispanic/Latino (H/L) and non-Hispanic Black (NHB) patients, have poor outcomes relative to non-Hispanic White (NHW) patients (16–18), as well as known differences in genetic drivers (19–22). Cancer disparities are the product of a complex range of socioeconomic, cultural, and biological contributors, of which genomic differences are one factor. The unique biology of many groups has not been adequately characterized because most research resources are built from participants who are NHW with European ancestry (6). A population that is often underrepresented in biological data sources is H/L patients with cancer (2, 4, 6, 7, 12, 23–25). For example, The Cancer Genome Atlas (TCGA), one of the most used cancer databases in research, contains data from approximately 11,000 tumors from 33 cancer types. Yet only approximately 18% of the sequenced tumors, combined, are from underrepresented groups, including only 3% with H/L ethnicity (1). Although ethnicity reporting is improving (10), the number of H/L patients is similarly low with respect to patients with breast cancer. For example, Ding and colleagues (26) noted that only 3.6% of patients with breast cancer (39 of 1,096) reported H/L ethnicity. ClinicalTrials.gov did not require the reporting of race/ethnicity information (if even collected) during result submission for trials until 2017 (27). Some previous publications describing TCGA resources do not even acknowledge H/L or other minority group patients as distinct, instead generalizing them into “other” or “not applicable” categories (28, 29). Even though the TCGA data describe many molecular changes that occur in cancer and have contributed to notable advances in treatment modalities, the data have also widened the gap in understanding molecular traits associated with ancestry-related differences among minority groups (1). Therefore, molecular changes and alterations described so far may not be as relevant in ethnically and racially diverse groups. Additionally, even within the studies that have H/L participants, these patients are often reported as aggregates or homogenous populations, further masking significant differences that can have an impact on their somatic mutation landscape.

As a part of the partnership between Ponce Health Science University in Ponce, Puerto Rico, and Moffitt Cancer Center (MCC) in Tampa, Florida, the Puerto Rico BioBank (PRBB; ref. 14) emerged in 2008 as a biospecimen resource that works to collect, process, and distribute tissues mostly from Puerto Rican individuals, as well as demographic and clinical data for use in cancer research. Its goal is to fill the gap in molecular data needed for translational studies that have an impact on H/L women, specifically the population of Hispanics in Puerto Rico (HPR). Most studies indicate that H/L and NHB women with breast cancer have significantly poorer survival outcomes (30–35) relative to NHW women. However, these findings are not universal, and some studies find that certain Hispanic groups have better prognostic outcomes than NHW women with breast cancer (31–52).

In Puerto Rico, breast cancer has been the most frequently diagnosed neoplasia in women and the cancer type with the highest mortality (50). Additionally, patients with breast cancer in Puerto Rico experience a worse 5-year survival rate after diagnosis that, so far, has not been directly associated with IHC surrogate markers, when compared with NHWs (52). After multivariate adjustments, Ooi and colleagues (31) showed that Puerto Ricans have significantly higher HRs than other H/Ls despite being given appropriate treatment. Puerto Rican patients with breast cancer in the United States are usually grouped in a broad Hispanic cohort, along with women from other very diverse H/L origins [including other Caribbean Hispanics (Dominican and Cuban), Central American Hispanics (including Mexican), and South American Hispanics and Latinos], generalizing the group as homogenous. However, some differences in risk factors among Puerto Rican patients with breast cancer in the United States, other Hispanic ethnicities, and Puerto Rican patients with breast cancer in Puerto Rico have been noted, warranting further investigations (51).

**Figure 1:**
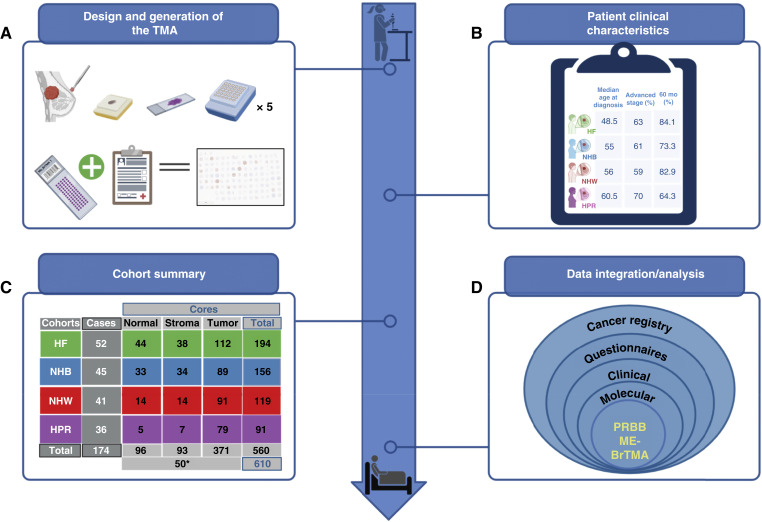
The multiethnic breast cancer cohort study. This study represents a long-term commitment of the PRBB to address factors that drive poor breast cancer outcomes within understudied populations. **A,** The ME-BrTMA was constructed from available formalin-fixed, paraffin-embedded breast tissues of well-annotated, consented study participants from the MCC-TCC and the PRBB. **B,** General patient clinical characteristics of the four cohorts (more details available in Supplementary Table S1). The TMA represents four groups: HF, HPR, NHB, and NHW. **C,** The final cohorts as represented by cores on the TMA include 45 NHB, 52 HF, and 41 NHW patients from the MCC and 36 HPR patients from the PRBB. The TMA is composed of five blocks with 610 total cores: 560 total cores from breast tissue (96 normal, 371 tumor, and 93 stromal cores) and 10 control cases on each block (50*). See Supplementary Table S3 for details. **D,** A representation of the levels of data that we seek to obtain as part of the broader study. Created in BioRender.com. Gordian, E. (2025) https://BioRender.com/l83r702 with additional edits in PowerPoint.

In previous work (53), we leveraged cancer registry and patient questionnaire data to describe four cohorts of patients with breast cancer from Florida and Puerto Rico. Herein, we use available tissue blocks representing a subset of patients from the previously described cohort (53) to construct a multiethnic breast cancer tissue microarray (ME-BrTMA). The ME-BrTMA contains samples from NHW, NHB, and Hispanic patients in Florida (HF), as well as from HPR patients. Supplementary Tables S1 and S2, within the Supplementary Information summarize the clinical and outcome characteristics of each cohort. Figure 1 highlights the construction of the ME-BrTMA (Fig. 1A) and how it fits into our larger goal to characterize (Fig. 1B) four cohorts of patients with breast cancer (Fig. 1C) at five levels (Fig. 1D), including cancer registry data (53), patient questionnaire data (53), clinical data, molecular data (in progress), and at the level of a representative TMA, allowing for spatial proteomic analysis using multiplex immunofluorescence. It is our intention that this TMA will support hypothesis-driven biomolecular research to understand breast cancer outcomes while representing multiple racial and ethnic groups.

## Materials and Methods

### Ethics statement

This is to certify that the Institutional Review Board (IRB) has reviewed and approved the research protocol titled “The Puerto Rico Biobank–PRBB” (IRB#00033379, Advarra IRB#00000971). The study has been evaluated for its ethical considerations, potential risks, and benefits to participants. The approval is granted based on the adherence to ethical guidelines and regulatory requirements of the U.S. Common Rule.

### The TMA

Tissue samples in the form of surgical blocks were collected from female patients with primary breast cancer diagnosed between 1998 and 2019, as well as from non–breast tumor controls and non–breast cancer healthy controls. Patients were selected from four race and ethnicity cohorts, defined as follows: NHW patients were consented to the Total Cancer Care (TCC) at the MCC, donated a tissue block with tumor, and identified their ethnicity as “non-Spanish” and their race as “White.” NHB patients consented to the TCC at the MCC in Florida, donated a tissue block with tumor, and identified their ethnicity as “non-Spanish” and their race as “Black.” HF patients consented to the TCC at the MCC, donated a tissue block with tumor, and identified their ethnicity as a specified or unspecified Spanish origin. HPR patients consented to the PRBB in Puerto Rico and donated a tissue block with tumor. Some patient data were acquired from the Puerto Rico Central Cancer Registry (RRID: SCR_023507). It is important to note that the H/L patients in our cohorts may be of any race. Of 52 patients in the HF group, 42 self-reported to be of the White race. In the HPR group, 22 patients of 36 self-reported to be of the White race. The patients did not self-report being on any other races. All tissue samples were collected after written informed consent under IRB-approved banking protocols based on adherence to ethical guidelines and regulatory requirements of the U.S. Common Rule. The collection of the HPR cohort by the PRBB was covered by IRB#00033379 and IRB#00000971. NHW, NHB, and HF patient samples were acquired under the TCC Protocol (NCT03977402; Advarra IRB# Pro00014441).

Because their numbers were limiting, blocks from NHB and HPR patients were identified first and all were used. Blocks from White patients, available in excess, were then matched to NHB and HF patients by age at diagnosis, body mass index, and pathologic T stage to the extent that the available data and tissue samples allowed. Among matched NHW patients, those with the most available tumor tissue were selected. An experienced breast cancer pathologist used fresh hematoxylin and eosin slides to mark the blocks for punching 1.0-mm cores of stroma, normal tissue, and two or more tumor cores. All tumor punches were performed from areas of greater than >30% cellularity. Treatment status was unknown prior to block selection. As was possible, two tumor cores, one stroma core, and one normal core were acquired from each patient, though there was variation in the amount and type of available tissue, and 81 patients are represented by three or more tumor cores. The ME-BrTMA was assembled in five individual blocks, with 135 to 150 tissue cores per block in the MCC Tissue Core Facility (RRID: SCR_012364). Randomization was performed among 610 cores across five blocks to avoid batch effects. Of the tissue cores included within each block, six are non–breast tumor controls (ovary, colon, pancreas, prostate, kidney, and brain) and four are non–breast normal tissue controls (kidney, salivary gland, colon, and tonsil). See Supplementary Table S3 for an overall summary of the 610 tissue cores of the TMA.

### Staining and analysis of the TMA

The ME-BrTMA was stained for hematoxylin and eosin, estrogen receptor (ER), progesterone receptor (PR), HER2, and Ki-67. The specific antibodies used were as follows: (i) ER (Ventana, cat. # 790-4324, RRID: AB_2857956), (ii) PR (Ventana, cat. # 790-2223, RRID: AB_2335976), (iii) Ki-67 (Ventana, cat. # 790-4286, RRID: AB_2631262), and HER2 (Cell Signaling Technology, cat. #4290, RRID: AB_10557104). Additional technical details are included in Supplementary Table S4, and detailed results for the 371 tumor cores are available in Supplementary Table S5. Scoring for ER/PR and HER2 was interpreted using the current clinical guidelines of the American Society of Clinical Oncology and College of American Pathologists. The five blocks of the TMA were scored individually by a clinical pathologist (author Marilin Rosa) who was blinded to the cohort (race/ethnicity grouping) identification. The ER was considered negative with less than 1% positively staining nuclei, low-positive with 1% to 10% positively staining nuclei, and positive with greater than 10% positively staining nuclei. For PR, samples with less than 1% positively staining nuclei were considered negative, and those with 1% or greater positively staining nuclei were considered positive. HER2 0 and 1+ were considered negative, HER2 2+ was considered equivocal, and HER2 3+ was considered positive (54, 55). For HR status, only cases with both ER and PR status were considered; HR− were ER− and PR− cases, and all others (ER–low-positive or ER-positive with a PR-positive result) were HR+. Clinical ER, PR, and HER2 results were acquired from cancer registry data sources to ensure that the tissue samples within the ME-BrTMA adequately represented the corresponding patient’s disease. Clinical HR was determined with only cases with both clinical ER and clinical PR status. To quantify the frequency of HR/HER2 subtypes, ER and PR were considered negative if the percentage of nuclear staining was less than 1% and positive with 1% or greater positive nuclei. HER2 was considered negative with a score of 0 or 1+, equivocal with a score of 2+, and positive with a score of 3+. Clinical HR/HER2 subtypes were determined with HER2 borderline or positive considered as HER2+.

### Statistical analysis

Statistical analysis of staining results was conducted using descriptive statistics, Fisher exact test, χ^2^ test, and Kruskal–Wallis test, as appropriate. Analysis was performed using R statistical software (version 4.2.0, R Core Team 2022; R Project for Statistical Computing, RRID: SCR_001905). χ^2^ tests were used to compare proportions, unless expected counts for categories were <5, in which case the Fisher exact test is used. All statistical testing and visualization code are publicly available at https://github.com/Moffitt-Cancer-Center/me-brtma-summary (GitHub, RRID: SCR_002630).

Comparison of ER, HR/HER2, and PAM50 subtypes were performed on categorical data by factor level using the Fisher exact test in R version 4.2.0. Comparisons of PR, HR, and HER2 were performed on categorical data using the χ^2^ test. Analysis of Ki-67 positivity (represented as continuous percentages) was compared among cohorts (NHW, NHB, HF, and HPR) using the Kruskal–Wallis H test in R version 4.2.0. No *post hoc* tests were used for subsequent evaluation.

### Gene expression analysis

Tumor RNA was acquired from patients with sufficient tissue samples for RNA extraction. RNA extraction was performed from a second formalin-fixed, paraffin-embedded block, where available. All RNA samples were DNase-treated and eluted in water. The samples were processed using Illumina TruSeq RNA Exome Kit and sequenced on one NovaSeq-600 S2-200 sequencing run (101 × 101). PAM50 gene expression signatures were calculated using the genefu package in R (56), and patients were classified according to their tumor-intrinsic molecular subtype into the following categories: basal-like, HER2-enriched, luminal A, luminal B, and normal-like.

### Data availability

All data are available within the main article and supplementary files. The data and analysis generated in this study and select TMA core images are available within this article’s Supplementary Tables S1–S15. Additional quality control analyses are present in Supplementary Tables S16–S21. Individual TMA and core images are available upon request from the corresponding author.

## Results

### The frequency of ER-negative and PR-negative expression is highest in tumor cores from patients from the NHB cohort

**Figure 2:**
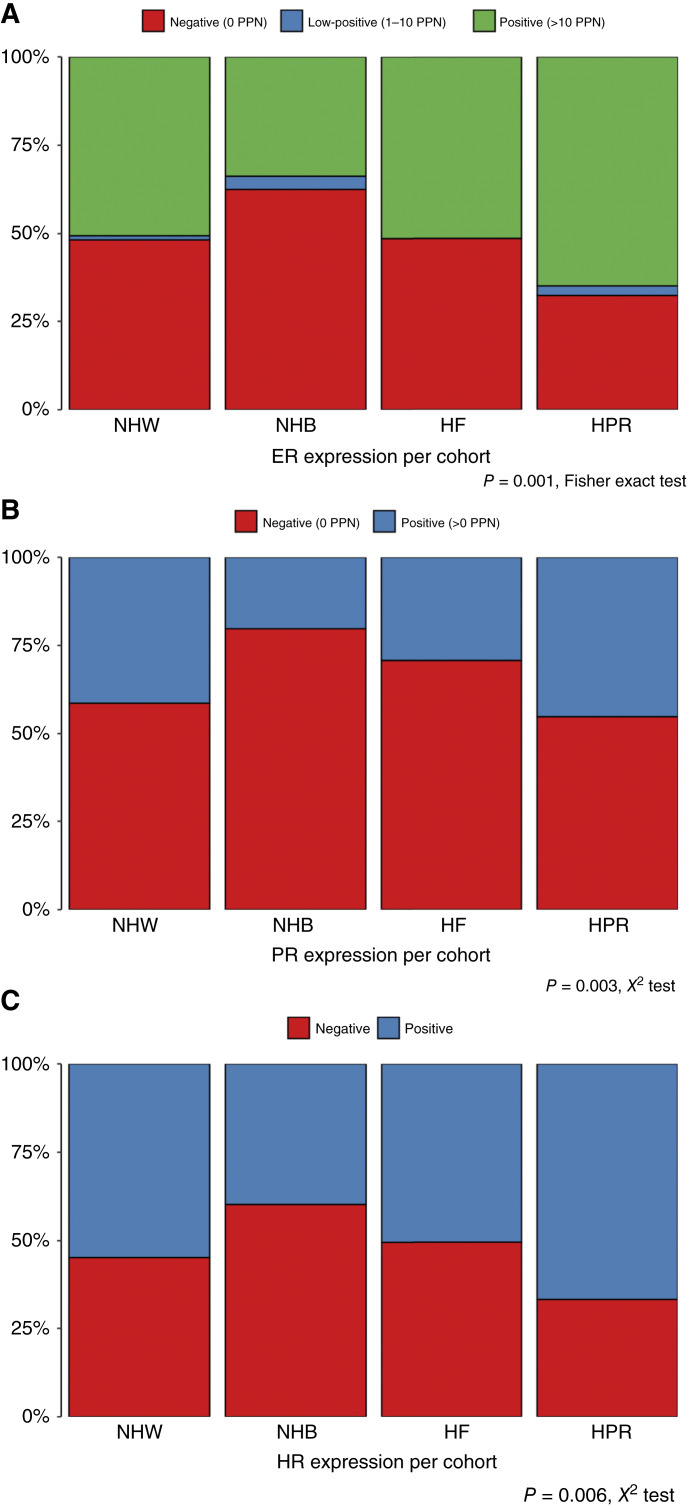
The frequency of ER-negative and PR-negative expression is highest in tumor cores from patients in the NHB cohort. **A,** Stacked bar chart showing that the frequency of negative ER expression is highest in cores from the NHB cohort (Fisher exact test, *P* = 0.001). **B,** Stacked bar chart showing that the frequency of PR-negative expression is highest in cores from the NHB cohort (χ^2^ test, *P* = 0.003). **C,** Stacked bar chart showing that the frequency of HR-negative expression is highest in cores from the NHB cohort (χ^2^ test, *P* = 0.006, χ^2^ test). PPN, percent positive nuclei.

The five blocks of the TMA were sectioned and stained with clinically relevant antibodies against ER, PR, HER2, and Ki-67 and were scored individually by a clinical pathologist (author Marilin Rosa). Supplementary Figures S1 through S4 provide examples of positively and negatively stained cores for each of the four antibodies from each cohort. To provide an initial characterization of the four cohorts represented on the ME-BrTMA, we compared the ER (Fig. 2A) and PR (Fig. 2B) expression among the cores of the four cohorts (Supplementary Table S6). The NHB cohort had the highest fraction of patients with ER-negative cores (63%, overall *P* = 0.001, Fisher exact test), PR-negative cores (80%, overall *P* = 0.003), and combined HR-negative (59%, overall *P* = 0.006) cores.

Using the determined ER and PR scores from individual cores, we sought to determine how well the values aligned with the patient-level clinical results. Supplementary Table S7 summarizes the results for the ER. For this analysis, we simplified the ER classification to positive or negative as the classification of ER stains as negative, low-positive, and positive is a new development for diagnostic purposes. Our existing clinical classifications do not include this level of resolution; therefore, we can compare the simplified ER stain results with the clinical ER status. Note that we also remove the equivocal from the clinical results. Of the 135 successfully evaluated cores for which we had documented ER-negative clinical results, 133 matched the expectations from the clinical reports. In contrast, two cores were low-positive. Of the 173 cores from ER-positive patients, 19 (11%) were ER-negative. Supplementary Table S8 summarizes the results of the PR cohort. Of the 165 cores from PR-negative patients, five cores (representing four patients) were scored positive. Of the 145 cores from PR-positive patients, 49 (34%) were scored negative in the TMA. Figure 2C and Supplementary Table S9 summarize the results for the combined ER/PR (HR). Of the 126 cores from HR-negative patients, four (3%) were positive, with ER and/or PR expressing 1% or greater positive nuclei. Of the 178 cores from clinically HR-positive patients, 25 (14%) were negative.

### Triple-negative (HR−/HER2−) is more frequently found in the NHB cohort

**Figure 3:**
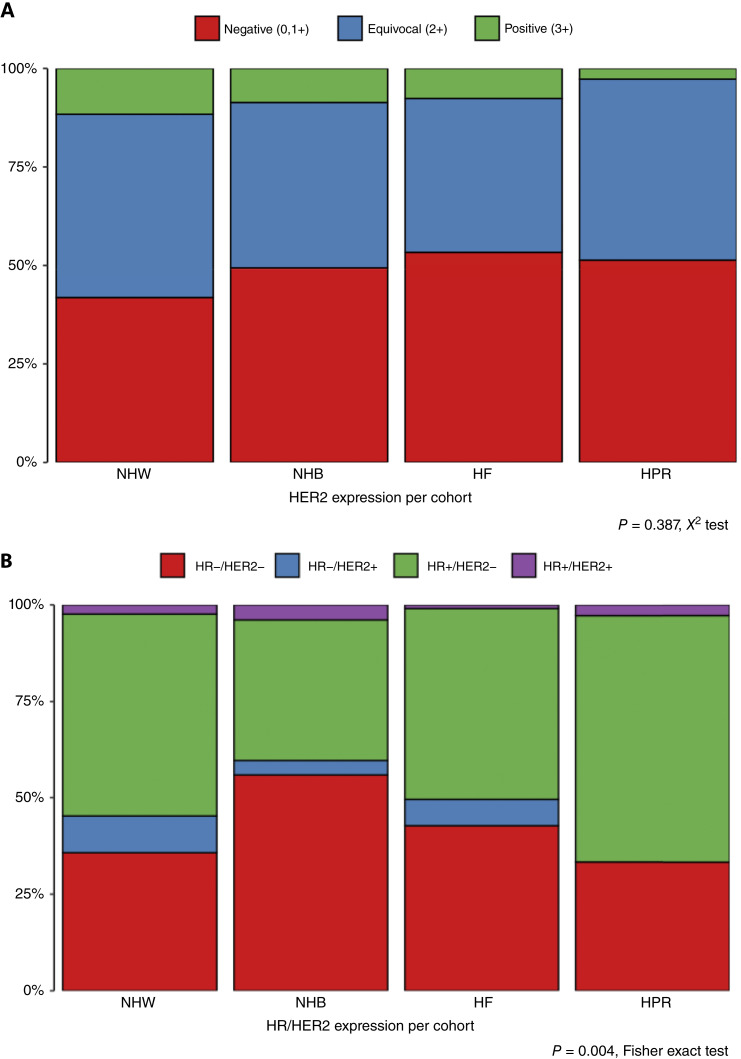
TNBC is most common in the NHB cohort. **A,** Stacked bar chart showing the fraction of patients with each level of HER2 expression among the four cohorts (*P* = 0.387, χ^2^ test). **B,** Stacked bar chart showing that the HR−/HER2− (triple-negative) phenotype is more frequent in the cores from the NHB patients (*P* = 0.004, Fisher exact test). HR, hormone receptor (ER and PR); PPN, percent positive nuclei.

The expression of the HER2 receptor is a critical predictive and prognostic clinical biomarker in breast cancer, and thus we stained our TMA with an HER2 antibody to estimate HER2 expression using published criteria (54). Supplementary Figure S3 highlights examples of HER2-stained cores from each cohort that were scored as HER2 0, HER2 1+, HER2 2+, or HER2 3+. Figure 3A shows that the frequency of HER2+ staining is similar by cohort with no statistically significant differences noted (*P* = 0.387, χ^2^ test). Supplementary Table S10 shows that of the 182 cores from HER2-negative patients, all were scored as either negative (*n* = 103, 57%) or equivocal (*n* = 79, 43%). Among 35 cores from HER2+ patients, 20 (57%) were scored as HER2 3+, and 15 were scored as negative (*n* = 8, 23%) or equivocal (*n* = 7, 20%). Patients with negative (HER2 0+) and positive (HER2 3+) staining have a straightforward therapeutic implication when compared with equivocal or the most recent clinical subclassification of HER2-low (IHC 1+ or IHC 2+ *in situ* hybridization nonamplified; ref. 57). Disagreements with whole slides stained for clinical purposes and core-based scoring are expected because the core size is significantly smaller and, therefore, less representative than the complete pathologic samples.

Figure 3B highlights our findings after aggregating ER, PR, and HER2 scores into distinct HR/HER2 subtypes; a significant difference in the frequency of each subtype by cohort was noted (*P* = 0.006, Fisher exact test, see Supplementary Table S11). The aggressive HR−/HER2− (triple-negative) disease comprised more than half (56%) of breast tumor cores from the NHB cohort, demonstrating a higher frequency of this subtype than in any other cohort (Fig. 3B). The HR+/HER2− subtype, which tends to be less aggressive and more readily treatable, was most frequently found among HPR patients (64%). Of the 80 cores associated with HR−/HER2− clinical results, 77 were also HR−/HER2− on pathology reading (Supplementary Table S12).

### High expression of the proliferative marker Ki-67 is more frequent among cores from the NHB cohort

**Figure 4:**
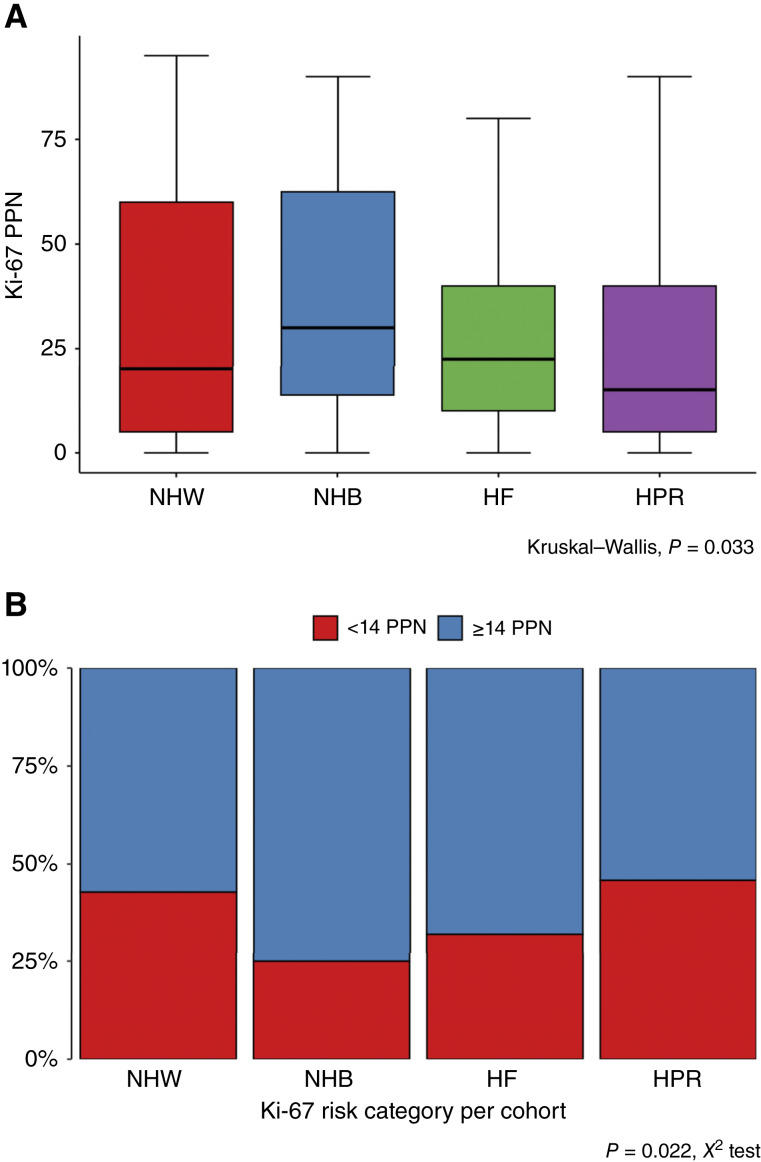
High expression of the proliferative marker Ki-67 is more frequent among cores from the NHB cohort. **A,** Boxplots comparing Ki-67% positivity as a function of the cohort. **B,** Risk categories for Ki-67 (low risk, <14 PPN vs. high risk >14 PPN) are shown as stacked bar charts. Supplementary Table S13 tabulates these data (see rows Ki-67 PPN and Ki-67 risk category). PPN, percent positive nuclei.

Ki-67 is a strong proliferative marker in breast cancer and can distinguish between the luminal A and the more aggressive luminal B subtype. Supplementary Figure S4 shows examples of Ki-67–stained cores from each cohort by high and low staining. For Ki-67, we chose a high-to-low risk cutoff of ≥14% Ki-67+ nuclei, based on previous reports of its demonstrated prognostic significance (58, 59). There was a significant difference (*P* = 0.033, Kruskal–Wallis test, statistic = 8.74, 3 degrees of freedom) in Ki-67 expression observed among the four cohorts. Fig. 4A shows the increased frequency of high expression of this proliferative marker among the NHB women in our study relative to the NHW, HF, and HPR women. Figure 4B demonstrates the fraction of patients in each cohort above and below the 14 percent positive nuclei cutoff. Supplementary Figure S5 and Supplementary Table S13 extend the analysis of Fig. 4, summarizing Ki-67 percent positivity by clinical subtype and by cohort.

### PAM50 analysis shows that the basal-like subtype is more frequent in the NHB patient cohort compared with the Hispanic cohorts

**Figure 5:**
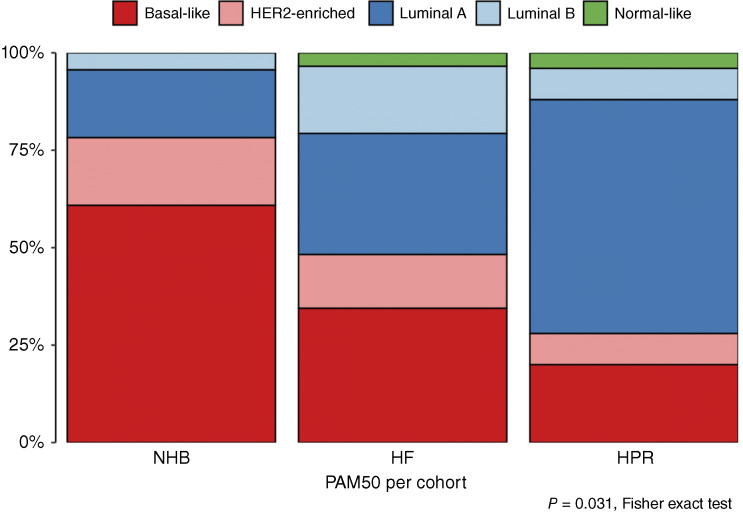
PAM50 analysis shows that basal-like subtype is most frequent among NHBs. Stacked bar chart shows the results of PAM50 gene expression analysis on patients represented on the ME-BrTMA with available RNA sequencing data. The data show that the basal-like subtype is most frequent among NHBs represented on the TMA (*P* = 0.031). Supplementary Table S15 tabulates the PAM50 subgroup breakdown by cohort. Note that RNA sequencing data for the NHW patients in cohort 1 are not included because they are not available.

On comparison of the PAM50 signature results by cohort, a significant difference (*P* = 0.031, Fisher exact test) in subtype frequency was observed. These data represent a subset of ME-BrTMA patients with adequate duplicate blocks available to us for RNA extraction and do not include any women from the NHW cohort. The aggressive basal-like subtype is more frequently found among NHB patients (61%) than among those from the HF (34%) and HPR (20%) cohorts. The 61% basal-like breast cancer observed in NHB women is close to the 56% triple-negative breast cancers (TNBC) observed in this cohort (Fig. 5; Supplementary Table S14). Though basal-like breast cancers and TNBCs share many features, not all basal-like subtypes are TNBCs (60). Therefore, the ∼5% difference observed in this cohort could represent additional molecular traits that can further help elucidate its aggressive phenotype. Luminal A subtype, which has a better prognosis than other subtypes, is more frequently found in the HPR cohort (60%) than in the HF (31%) and NHB (17%) cohorts. Supplementary Table S15 provides a summary of PAM50 subtypes by HR/HER2 status and cohort.

### Additional quality control analysis demonstrates that the tissue cores of the ME-BrTMA represent the patients included

Two additional quality control analyses were performed to demonstrate that the ME-BrTMA is representative of the patients included, as recommended by the reviewers of this article. Supplementary Tables S16–S21 examined ER, PR, and HER2, respectively. In the first approach, we examined how well high and low individual tumor scores matched among all patients. In such tabulations, the largest values fall along the diagonal, indicating consistency in scoring. Supplementary Tables S16, S18, and S20 reveal that in every case, there is excellent consistency. However, as expected, when 1-mm cores are used to represent an entire tumor, there are discrepancies. ER consistency was the best with only 5 discrepancies, PR had 15 discrepancies, and HER2 had 30 discrepancies. In the second approach, we tabulated how many cores represent each patient, broken down by cohort. Supplementary Tables S17, S19, and S21 reveal that 45% of the patients are represented by three or more tumor cores, 30% are represented by two cores, 24% are represented by one core, and 2.4% are not represented by tumor core because of manufacturing errors.

## Discussion

Through future studies performed by our colleagues and investigators in the community, the ME-BrTMA represents a tool for diversifying translational breast cancer research. Previous TMAs (13, 61) have allowed us to detect levels of mitotic kinases in breast cancer tissues of patients of different races and ethnicities and correlate their levels with pathologic subtypes of breast cancer (e.g., PR, ER, HER2, and Ki-67), as well as surrogate markers of metastasis (e.g., vimentin). In addition to the utility inherent to a TMA, the ME-BrTMA is noteworthy because of the racial and ethnic diversity of patients from whom tissue was acquired, the robust patient-level data available, and its representation of known biological differences in the four groups.

The expression of the ER and PR hormone receptors is a critical prognostic and predictive clinical biomarker in breast cancer. Patients with ER/PR+ breast cancers are candidates for endocrine therapy with tamoxifen as adjuvant therapy. The National Comprehensive Cancer Network guidelines currently recommend tamoxifen or aromatase inhibitors for postmenopausal patients with breast cancer as an option to prevent recurrence and improve survival. However, clinical trials have shown that breast cancer recurrence after 5 years of adjuvant endocrine therapy can range from 10% to 41% (32), suggesting the need to further stratify patient responses, considering their unique tumor biology along with their genetic ancestry. In addition to ER and PR, HER2 testing also guides treatment options for patients with breast cancer. Several decades back, HER2+ tumors were considered aggressive as TNBC. However, the discovery of mAb treatments, such as trastuzumab, in the early 1980s changed the outcome of early diagnosed patients and patients that exhibit HER2+, *de novo* metastatic disease (62).

We have previously analyzed HR/HER2 expression in broader cohorts of NHW, NHB, HF, and HPR patients and found the prevalence of HR−/HER2− disease in our sample to reflect the frequency at which this subtype has been found in the literature (53). However, the patients included within the ME-BrTMA represent a sample of tumors with a higher frequency of HR−/HER2− than we may have expected (33%–56%), given that only 12% to 27% of patients in each race and ethnicity group in our previous study were HR−/HER2−. Additionally, although we previously found a significantly lower frequency of HR+/HER2− disease in HPR patients relative to NHW patients, this subtype was most frequently found among HPR patients within the ME-BrTMA compared with each of the other three cohorts. We expect that these differences in frequency relate to the fact that patients with aggressive disease are more likely to seek treatment at academic hospitals and are more likely to consent to research protocols than patients who are represented in cancer registries.

Clinical, pathologic, and molecular data obtained from investigations using the ME-BrTMA will also provide information on another crucial factor affecting these groups: treatment response. It is important to highlight that limited understanding is available about the susceptibility to chemotherapy agents among certain populations. Previous reports have addressed the importance of finding ethnicity-specific genes or gene signatures that can explain response, resistance, or toxicities to cancer compounds such as alkylating agents or mAbs (trastuzumab) used for treating breast cancer in HER2+ patients (8, 9).

As a measure of proliferation, Ki-67 expression can be used for breast cancer prognosis and to predict response to chemotherapeutic drugs. This marker is especially useful in the setting of HR+/HER2− disease because it can be used to differentiate between luminal subtypes (63). However, its use has not been completely adopted as standard of care in pathologic reporting because of the inconsistencies in surgical acquisition, IHC analysis, and cutoff values. Having said that, Ki-67 is considered an informative marker that can aid in prognosis as well as clinical decisions if shown to improve these in conjunction with other molecular markers (64). The degree of Ki-67 expression has been seen to vary by racial/ethnic group, with significantly higher Ki-67 scores for NHB patients relative to NHW patients (65).

To begin focusing on the unique biological features of each minority group, the PAM50 panel results were analyzed for a subset of the NHB, HF, and HPR groups. NHB and HPR patients show an inverse expression of the most and least aggressive subtypes, with most tumors from NHB patients being of the aggressive basal-like subtype and most tumors from the HPR patients being of the more favorable luminal A subtype. Previously, we noted a similarly poor 5-year overall survival in populations of NHB and HPR patients (53). In combination with this marked difference in PAM50 subtypes between NHB and HPR patients in the ME-BrTMA, these findings warrant further investigation into the biological and social drivers of poor survival among HPR patients.

As additional data resources become available, we aim to further characterize the patients from whom ME-BrTMA tissues were acquired. Genetic ancestry analysis will allow us to better represent the relationship between various biological markers and a patient’s heritage, without relying on reported race and ethnicity as proxies for true ancestry. We will also have a broader array of clinical data, particularly about treatment regimens and timing, because these contribute to patient prognosis and, in neoadjuvant chemotherapy, can affect the ME-BrTMA tissue samples. Continuous improvements are being made in data sharing and accessibility to encourage further utilization of this racially and ethnically diverse resource.

In conclusion, the unique breast tumor biology of Puerto Rican patients represents a significant gap in the breast cancer literature. We have created a diverse, well-annotated resource to support investigators in filling this gap through translational breast cancer research. As one component of a long effort by the PRBB, the ME-BrTMA allows the opportunity to explore the tumor biology of Puerto Rican patients with breast cancer in the context of larger-scale trends in disease prognosis that we have observed.

## Supplementary Material

Figure S1 Supplementary Figure 1

Figure S2 Supplementary Figure 2

Figure S3 Supplementary Figure 3

Figure S4 Supplementary Figure 4

Figure S5 Supplementary Figure 5

Table S1 Supplementary Table 1: Analysis of basic clinical and data regarding each cohort.

Table S2 Supplementary Table 2: Outcomes for ME-BRTMA by cohort.

Table S3 Supplementary Table 3: Summary of all 610 cores of the TMA.

Table S4 Supplementary Table 4: The antibodies used for immunohistochemistry.

Table S5 Supplementary Table 5: IHC scoring values of the 371 tumor cores on the TMA.

Table S6 Supplementary Table 6: Analysis of all IHC scoring values of the 371 tumor cores on the TMA by cohort.

Table S7 Supplementary Table 7: Comparison of Clinical ER calls vs. TMA stain evaluations (simplified).

Table S8 Supplementary Table 8: Comparison of Clinical PR calls vs. TMA stain evaluations.

Table S9 Supplementary Table 9: Comparison of derived Clinical HR calls vs. derived TMA stain evaluations.

Table S10 Supplementary Table 10: Comparison of Clinical HER2 calls vs. TMA stain evaluations.

Table S11 Supplementary Table 11: Summary of HR/HER2 stain evaluation by cohort.

Table S12 Supplementary Table 12: Comparison of derived Clinical HR/HER2 calls vs. TMA stain evaluations.

Table S13 Supplementary Table 13: Summary of risk categorizations Ki-67 across cohorts.

Table S15 Supplementary Table 15: Summary of PAM50 types by HR/HER2 status and cohort.

Table S14 Supplementary Table 14: Summary of PAM50 by cohort.

Table S16 Supplementary Table 16: Agreement of ER scores of tumor cores for same patient in TMA.

Table S17 Supplementary Table 17: Summary of number of ER-stained cores per patient (by cohort)

Table S18 Supplementary Table 18: Agreement of PR scores of tumor cores for same patient in TMA.

Table S19 Supplementary Table 19: Summary of number of PR-stained cores per patient (by cohort).

Table S20 Supplementary Table 20: Agreement of tumor cores HER2 scores for same patient in TMA.

Table S21 Supplementary Table 21: Summary of number of HER2-stained cores per patient (by cohort).
