# Supplementary material for: Creation and Characterization of a Breast Cancer Tissue Microarray Including Black and White Patients from Florida and Hispanic Patients from Puerto Rico and Florida
Source: Cancer Res Commun. 2025 May 16;5(5):804–13. doi: 10.1158/2767-9764.CRC-24-0650 (PMC12082392; doi:10.1158/2767-9764.CRC-24-0650)
Supplement: Figure S2 — Supplementary Figure 2 [file crc-24-0650_figure_s2_suppsf2.pdf]

Supplementary Figure 2

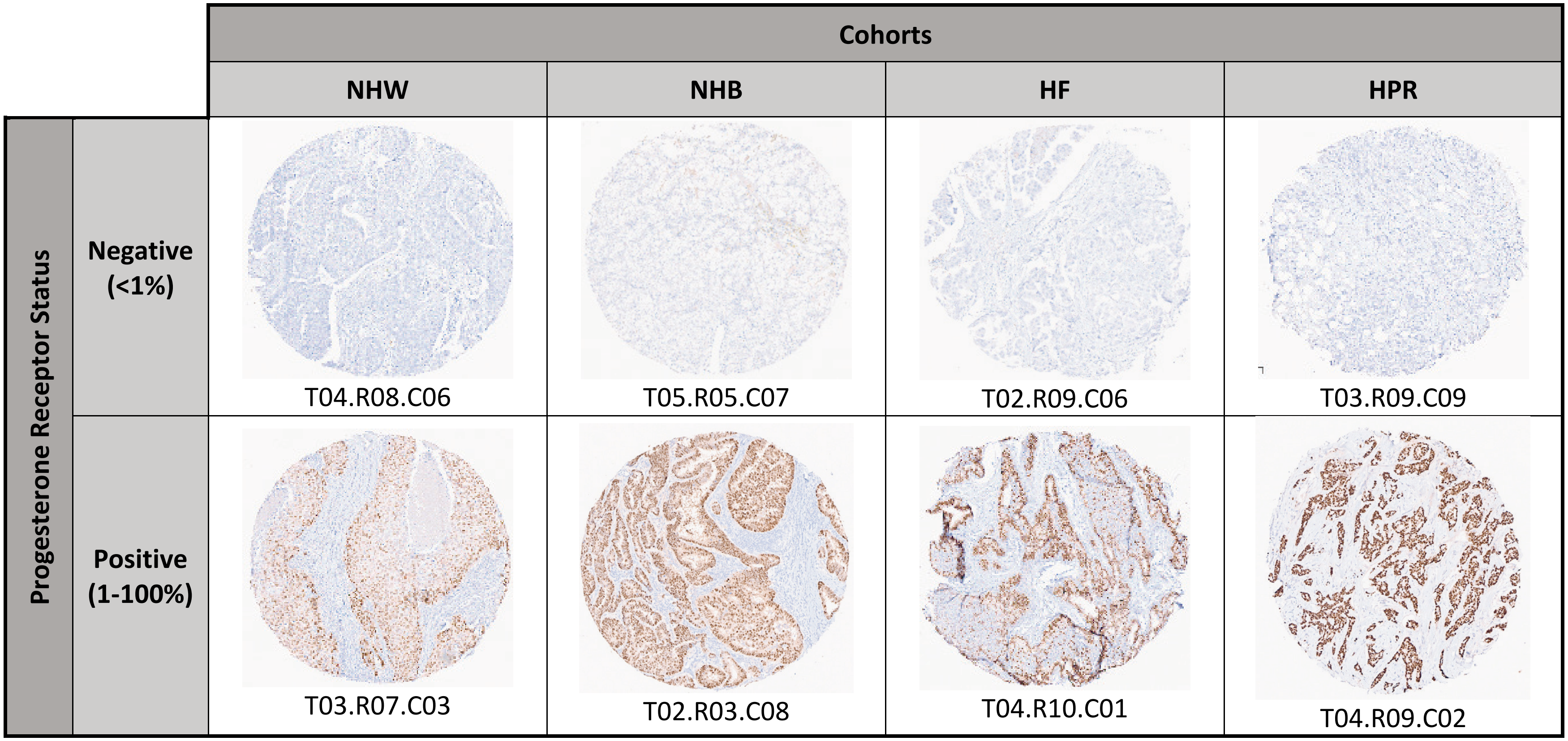

Supplementary Figure 2. Expression of the progesterone receptor in example cores by cohort and staining pattern. Examples of cores from each cohort stained for ER. Top, cores which were considered negative (<1% positive). Bottom, cores which were considered positive (1-100%). Examples of low positive cores were not included as examples due to their rarity.
