# Supplementary material for: Creation and Characterization of a Breast Cancer Tissue Microarray Including Black and White Patients from Florida and Hispanic Patients from Puerto Rico and Florida
Source: Cancer Res Commun. 2025 May 16;5(5):804–13. doi: 10.1158/2767-9764.CRC-24-0650 (PMC12082392; doi:10.1158/2767-9764.CRC-24-0650)
Supplement: Figure S3 — Supplementary Figure 3 [file crc-24-0650_figure_s3_suppsf3.pdf]

Supplementary Figure 3

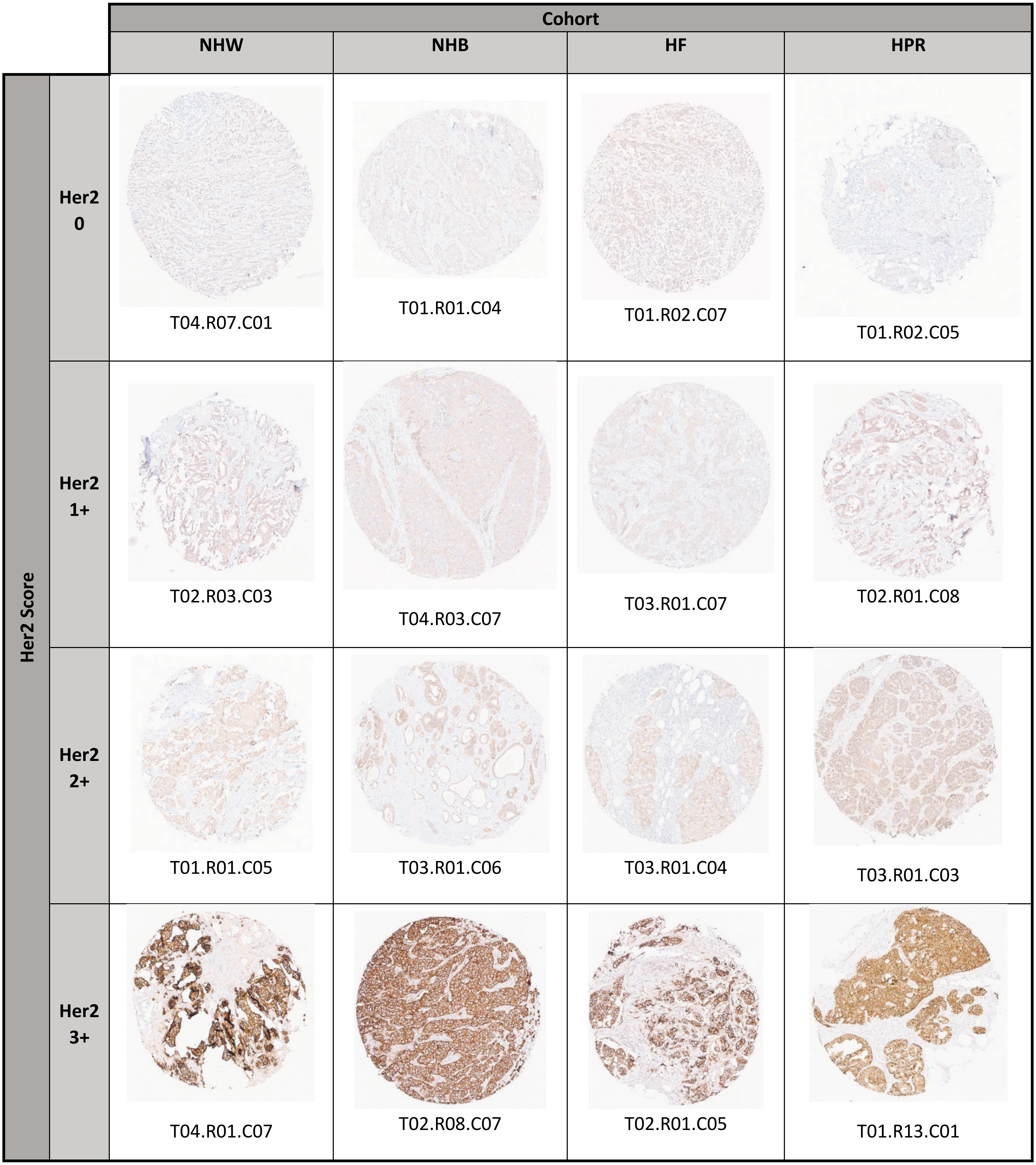

Supplementary Figure 3. Expression of HER2 is similar among the four cohorts. Examples of cores from each cohort stained for HER2. First row, cores which were considered HER2 negative. In the second, third and fourth rows are examples of cores which were scored +1, +2 and +3, respectively.
