# Supplementary material for: Creation and Characterization of a Breast Cancer Tissue Microarray Including Black and White Patients from Florida and Hispanic Patients from Puerto Rico and Florida
Source: Cancer Res Commun. 2025 May 16;5(5):804–13. doi: 10.1158/2767-9764.CRC-24-0650 (PMC12082392; doi:10.1158/2767-9764.CRC-24-0650)
Supplement: Figure S4 — Supplementary Figure 4 [file crc-24-0650_figure_s4_suppsf4.pdf]

Supplementary Figure 4

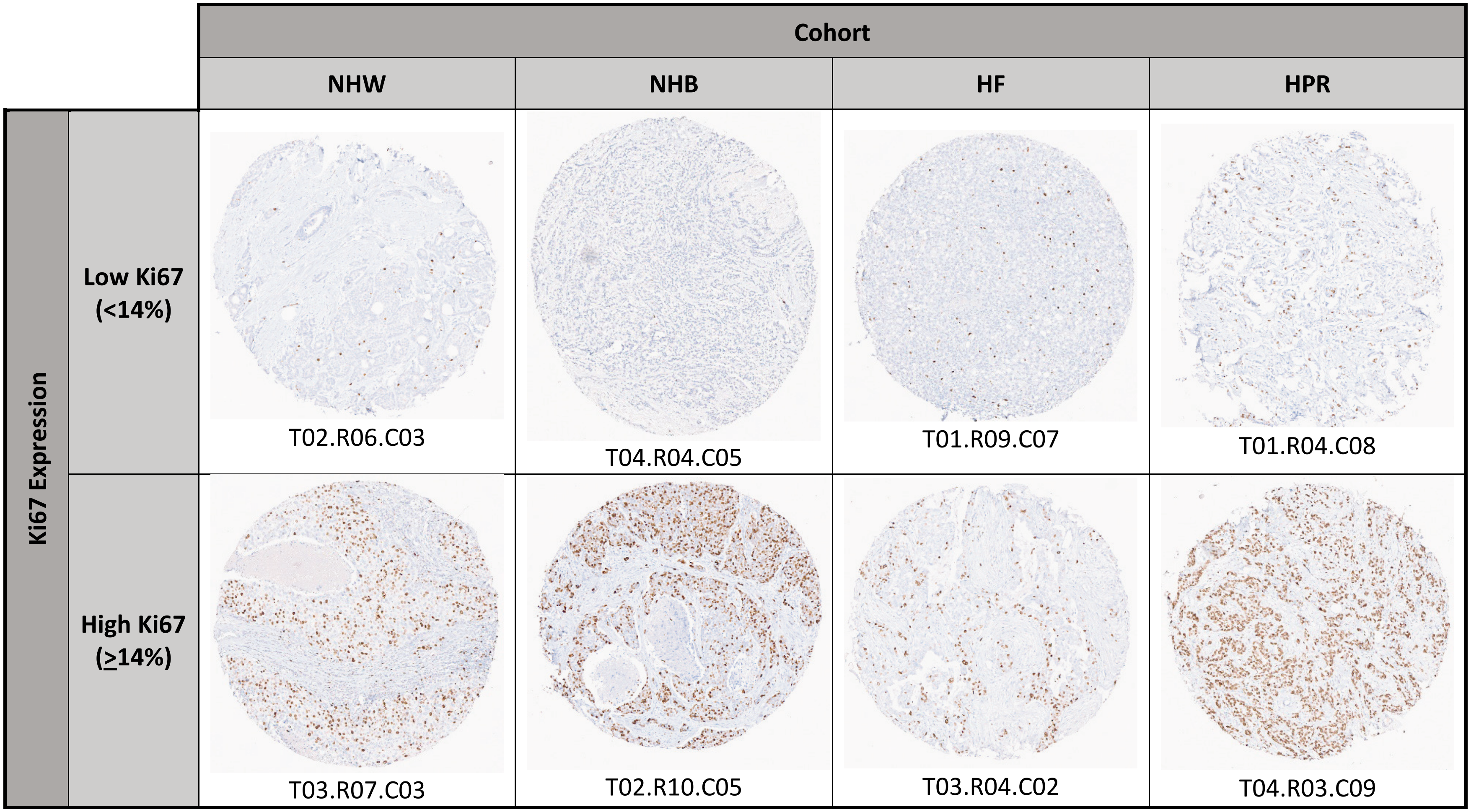

Supplementary Figure 4. Expression of the proliferative marker Ki-67 in example cores by cohort and staining pattern. Examples of cores from each cohort stained for the proliferative marker, Ki-67. Top, cores which were considered low in Ki-67 (<14% positive). Bottom, cores which were considered high in Ki-67 staining (≥14%).
