# Supplementary material for: Creation and Characterization of a Breast Cancer Tissue Microarray Including Black and White Patients from Florida and Hispanic Patients from Puerto Rico and Florida
Source: Cancer Res Commun. 2025 May 16;5(5):804–13. doi: 10.1158/2767-9764.CRC-24-0650 (PMC12082392; doi:10.1158/2767-9764.CRC-24-0650)
Supplement: Figure S5 — Supplementary Figure 5 [file crc-24-0650_figure_s5_suppsf5.pdf]

Supplementary Figure 5

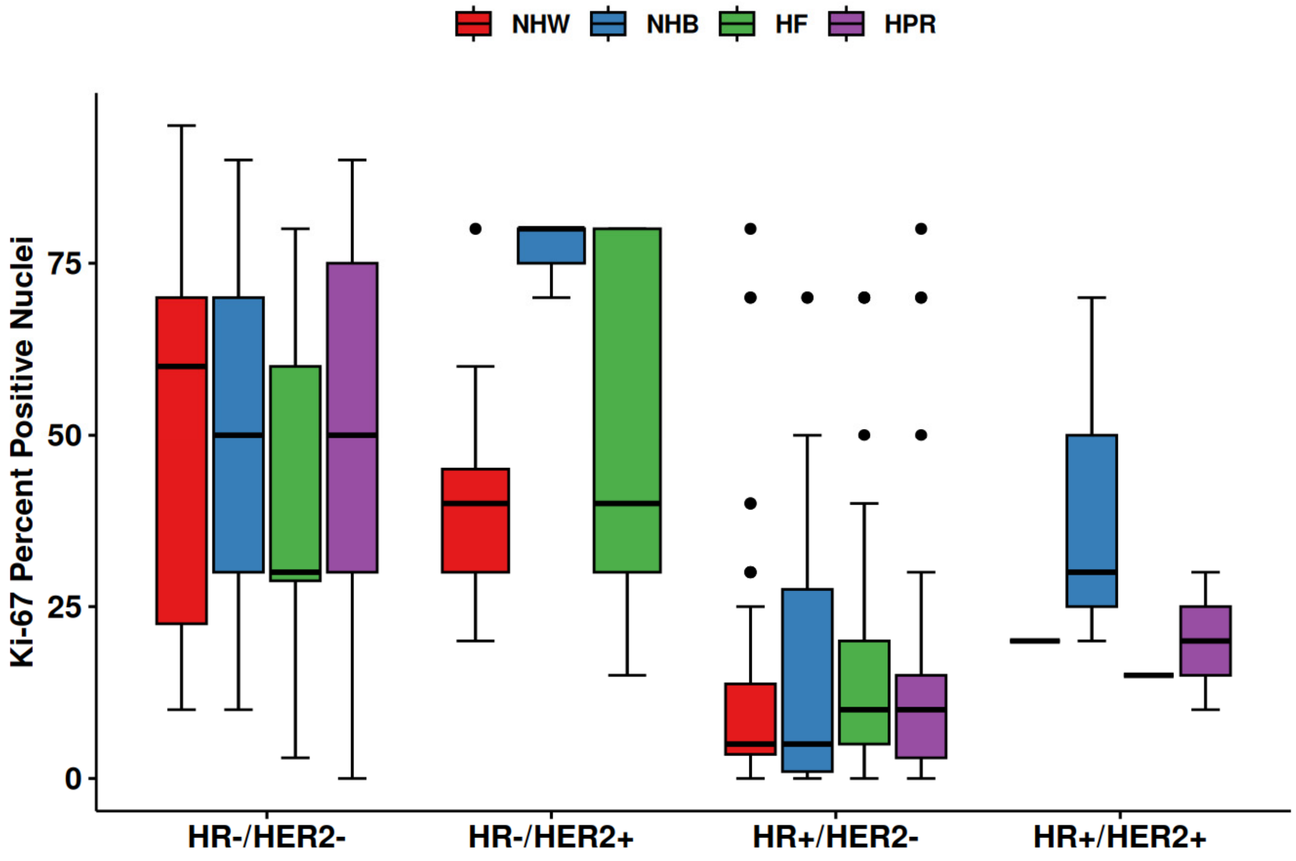

Supplementary Figure 5: Boxplot of Ki-67 percent positivity by clinical subtype and by cohort.
